# From Biosignals to Bedside: A Review of Real-Time Edge Machine Learning for Wearable Health Monitoring

**DOI:** 10.3390/bioengineering13050559

**Published:** 2026-05-15

**Authors:** Mustapha Oloko-Oba, Ebenezer Esenogho, Kehinde Aruleba

**Affiliations:** Centre for Artificial Intelligence and Multidisciplinary Innovations, College of Accounting, University of South Africa, Pretoria 0002, South Africa; esenoe@unisa.ac.za

**Keywords:** wearable biosignals, edge computing, real-time inference, multimodal sensor fusion, model compression, calibration and uncertainty quantification

## Abstract

Wearable devices increasingly capture biosignals such as electrocardiograms, photoplethysmograms, inertial signals, and electrodermal activity during daily life, enabling earlier detection and continuous monitoring outside the clinic. Real-time edge machine learning can convert these streams into timely, privacy-preserving inference by placing computation on a wearable (device-only) or a paired phone, with intermittent cloud assist used selectively for dashboards, summarisation, and lifecycle management. Clinical adoption remains uneven because free-living data are noisy, labels are often delayed, and device ecosystems evolve over time. This narrative review organises the literature as an end-to-end deployment pathway: sensing and artefact management, streaming windowing and multimodal alignment, and model families suited to on-device inference. We compare classical feature-based pipelines with learned representations, including compact CNN/TCN and recurrent and efficient attention-based models, and discuss when self-supervised pretraining and distillation are most useful in low-label settings. We then synthesise deployment engineering levers (quantisation, pruning, and distillation) and benchmarking requirements, emphasising runtime constraints that determine feasibility: latency per update, peak RAM, energy per inference, duty cycle, and thermal behaviour. Applications are grouped across cardiovascular monitoring, blood pressure and haemodynamics, sleep and respiration, and movement and stress, with explicit attention to false-alert burden, adherence, and workflow integration. To support translation, we provide a validation ladder and a reliability toolkit covering calibration, uncertainty-aware thresholds and deferral, drift monitoring triggers, and safe update governance. The novelty of this review is a deployment-oriented synthesis that ties modelling choices to edge tiers and resource budgets and provides reusable reporting templates, including an edge-cost card and comparative tables spanning modalities, models, deployment levers, applications, and reliability requirements.

## 1. Introduction and Scope

### 1.1. Why Edge ML for Wearables

Wearable health monitoring has moved routine measurement from the clinic into daily life. Contemporary devices can record biosignals such as electrocardiograms (ECGs), photoplethysmograms (PPGs), respiration surrogates, and motion continuously for days or weeks, which is valuable for intermittent or activity-dependent conditions that may be missed by brief spot checks [1,2,3]. This shift creates opportunities for earlier detection, longitudinal follow-up, and lower-burden monitoring, but it also exposes systems to motion artefacts, variable contacts, and changing contexts, which are less prominent in controlled recordings [1,4].

Real-time analysis is increasingly expected, particularly for cardiovascular screening and event detection, where delays can reduce clinical usefulness [4,5]. However, signal streams are generated at the point of measurement, while connectivity can be intermittent and energy budgets restricted. Uploading raw biosignals for cloud processing can increase latency, drain batteries through radio transmission, and widen exposure of sensitive data during transfer and storage. Edge computing, in which computation is placed close to data acquisition sites, directly targets these constraints and has been widely discussed as a practical fit for health monitoring [6,7]. Surveys of on-device inference and TinyML reinforce the idea that the choice between local inference and cloud inference is an architectural decision shaped by latency, memory, energy, and tolerance to connectivity loss, rather than an implementation detail [8]. Throughout this review we use the term privacy-preserving inferenceto reflect the practical advantage of limiting transmission of raw biosignals when local processing is feasible.

This is where the biosignals-to-bedside framing becomes important. The goal is not only to label a window of data, but to deliver an actionable output under device constraints and workflow constraints. Recent scoping work has noted that many real-time wearables studies do not report operational characteristics clearly, and that real-world validation remains limited compared with offline evaluation on public datasets [5]. Our contribution is a deployment-oriented synthesis that links modelling choices to concrete constraints and evidence requirements. We focus on how inference placement affects latency, battery use, and privacy exposure; which model families are commonly used under tight resource budgets; and which validation choices best support translation beyond the lab [7,8,9]. The intended audience includes bioengineers and device developers building sensing systems, clinical and rehabilitation engineers responsible for monitoring pathways, and computer scientists working on efficient learning and deployment [10].

### 1.2. Scope, Key Definitions, and Review Structure

This review focuses on wearable health monitoring where biosignals are analysed in real time using computation placed on a wearable device or a paired phone, with optional intermittent cloud assist for summarisation, dashboards, or lifecycle management. We prioritise ECG, PPG, inertial sensing, and electrodermal activity (with temperature where relevant) because these modalities dominate current ambulatory monitoring practice and represent distinct sensing failure modes that shape both modelling and deployment [1,11]. We include device-only and device-plus-phone designs, and we include edge-assisted patterns where the edge performs first-stage processing and transmits features or alerts when needed. We exclude cloud-only pipelines that require continuous upload of raw signals, because they operate under different bandwidth, privacy, and reliability constraints and lead to different failure modes [6,8].

We use real time to mean that the system produces outputs within a time window that is useful for the intended action, such as an alert, a prompt to repeat a measurement, or a recommendation for follow-up. Acceptable delay is therefore task dependent. We distinguish two common streaming styles: window-based inference, where short (often overlapping) segments are processed at a fixed cadence, and event-driven inference, where computation is triggered by changes in signal quality, detected episodes, or context. We also distinguish edge tiers, namely device-only processing versus device plus phone, because this choice strongly affects latency, memory limits, energy use, and update pathways [8,9]. When we refer to bedside, we mean decision support and monitoring pathways, not necessarily intensive care. This includes screening, triage, and longitudinal follow-up, where wearable outputs may inform a clinician, a service, or the individual, and where reliability can be as important as accuracy [4,5].

The review has three practical deliverables, which together define the novelty of the paper. First, we provide an end-to-end taxonomy that links sensing realities and signal quality to modelling choices and deployment constraints. Second, we synthesise model families through a deployment lens, focusing on when classical feature pipelines, compact deep models, and pretrained representations are most appropriate under edge budgets [8,9]. Third, we consolidate evaluation and reporting practices into a validation ladder and a reliability toolkit, motivated by evidence that operational characteristics and validation depth are often underreported in the real-time wearables literature [5]. We organise the paper around four axes that recur across studies: signal modality, clinical or health task, edge tier, and reliability requirements. Section 2 summarises biosignals, artefacts, streaming structure, and ground-truth realities. Section 3 reviews representations and model families for streaming inference. Section 4 covers deployment engineering, including compression, benchmarking, and edge architectures. Section 5 maps applications to validation designs and reliability practices. Section 6 synthesises open challenges, practical recommendations, and future directions.

## 2. Wearable Biosignals and Edge Data Realities

### 2.1. Core Modalities and Artefacts

Wearable edge systems most often rely on four sensing streams: electrocardiography (ECG), photoplethysmography (PPG), inertial sensing (accelerometer and gyroscope, often grouped as an IMU), and skin-facing signals such as electrodermal activity (EDA) and skin temperature. ECG and PPG are the main routes to cardiac timing and circulation-related endpoints, but they are not used “as-is”. They require post-processing to obtain clinically meaningful measures such as heart rate (HR), heart rate variability (HRV), and oxygen saturation (SpO_2_) [12,13]. IMU data provides the movement context that underpins step counts, posture and activity state, and gait parameters, and it can also serve as a reference stream for motion-related noise [14,15]. EDA, typically acquired as skin conductance response (SCR) or related measures, is widely used as a marker of sympathetic arousal, while skin temperature helps interpret thermoregulation and environmental exposure [14].

Each modality has characteristic failure modes, and these are amplified in wearables because the sensors are small, lightly coupled to the body, and exposed to daily life motion. Motion artefact is a dominant issue for both ECG and PPG, and it co-occurs with other disturbances such as power-line interference and false detections when the signal morphology is distorted [13,16]. For ECG, signal degradation is tightly linked to the electrode–skin interface. Changes in contact area, pressure, and hydration shift skin–electrode impedance and can lower the signal-to-noise ratio (SNR), especially during movement [17]. Beyond impedance alone, motion can also disrupt electrochemical equilibrium at the interface and change the thickness of the sweat layer, which in turn perturbs electrode capacitance and introduces baseline drift and parasitic voltages [18]. Similar mechanisms appear in capacitive ECG, where motion artefacts and circuit distortions can be confounded; recent reviews emphasise the need for explicit models and reference signals (for example, electrode–tissue impedance) to support effective suppression [19].

For PPG, motion artefact is particularly difficult because its spectral content overlaps the physiological band, so simple band-pass filtering often fails in active settings. In addition, baseline wander and parasitic current from ambient light can corrupt the measured waveform and reduce SNR [20]. Practical wearables therefore face a combined optical and mechanical problem: small shifts in sensor position, changes in contact pressure, and changing illumination conditions can all bias HR and SpO_2_ estimates [12,13,20]. IMU signals have a different set of artefacts: sensor placement and sensor-to-segment alignment vary across users and days, and this variability propagates into derived gait parameters and activity classifiers [15]. Temperature and EDA are also sensitive to interface conditions: changes in sweat volume and composition affect skin conductance, and electrode hydration and material choice can shift impedance and motion sensitivity over time [14,21].

Because downstream inference can only be as reliable as its input, most “biosignals-to-bedside” pipelines need an explicit signal quality stage before making or updating predictions. In the wearable ECG/PPG literature, this appears as signal quality indices (SQIs) or usability measures, where segments are classified as usable versus non-usable for a target endpoint (for example HR or SpO_2_) [13]. This is not a cosmetic step: simplistic artefact detectors can mistakenly reject clinically meaningful physiology (for example fast-changing HR dynamics or atrial fibrillation) as corrupted data [13]. In this review, we therefore treat artefact detection and SQI as prerequisite controls for edge deployment: they decide when to trust an estimate, when to defer, and when to request more data or an alternative modality [22]. Table 1 summarises the core wearable modalities, typical sampling ranges, dominant artefacts, and common preprocessing or SQI checks used in edge monitoring pipelines.

### 2.2. Streaming Data Structure

Wearable monitoring becomes a streaming problem as soon as the system is expected to update outputs continuously rather than produce a single summary at the end of a recording. In practice, choices such as window length, stride, buffering, and resampling are shaped by the sampling rates and artefact behaviour of each modality (Table 1). Most pipelines therefore reduce the stream into small analysis units using windowing. A fixed window has a duration (or sample length) and a stride. Overlap is introduced when the stride is shorter than the window length, so the model can refresh outputs more frequently than the window duration, at the cost of repeated computation [23]. This is why buffering is not optional. The device must retain the most recent window (often in a ring buffer) and, if overlap is used, it must retain enough history to avoid re-reading raw samples from slower storage.

Overlap interacts with the model architecture. For convolutional networks, successive overlapping windows contain many identical subsegments. Kechris et al. show that it is possible to reuse intermediate activations between overlapping windows by exploiting convolutional shift invariance, reducing the repeated work that overlap normally creates [23]. However, the same work also highlights a key complication for streaming deployments: operations such as zero padding and pooling are not shift-invariant, so naive reuse can introduce drift between streaming outputs and standard full-window inference [23]. In other words, the stream structure is partly determined by the model design, not only by the sensing task. A practical framing for this review is therefore to treat window definition (length, stride, and overlap) and model temporal operators (padding, pooling, and dilation) as a single co-design problem.

#### 2.2.1. Event-Triggered Inference: Design Patterns, Failure Cases, and Trade-Offs

A second primitive is event-triggered inference. Instead of running a full model at a fixed cadence, the system triggers heavier computation, storage, or transmission only when a condition is met. This reduces energy use and avoids unnecessary radio activity. A clear example is embedded atrial fibrillation screening where the device performs local inference and keeps wireless communication off, activating it only to transmit an alert when atrial fibrillation is detected [24]. In practice, event-triggered pipelines are commonly implemented as a two-stage pattern: a low-cost sentinel runs continuously (for example, SQI gating, a simple detector, or a small model) and triggers a higher-cost confirmatory stage only when needed. The trigger can be defined by (i) a threshold on a model score (e.g., probability exceeding τ), (ii) a change-point in features, (iii) a sustained run of suspicious windows (persistence logic), or (iv) a quality drop that requires re-acquisition. Event triggers can also operate within the stream scheduler, for example by temporarily shortening stride (higher update rate) during suspected events, or by gating which feature extractors run for a given segment [25].

Event triggers introduce specific failure cases that should be reported. Threshold triggers can amplify boundary effects when an event begins near the end of a window, leading to delayed detection unless overlap or persistence rules are used. Gating can also create blind spots if the sentinel is overly conservative or if SQI logic incorrectly flags clinically meaningful segments as unusable. A useful safeguard is to couple triggering to both confidence and quality: a high-risk score with low quality can be routed to re-measurement or a confirmatory modality rather than immediate alerting. The central trade-off is energy versus delay: aggressive triggering (low thresholds, short persistence) reduces time-to-detection but increases compute and alert burden, while conservative triggering saves energy but can miss short episodes or delay escalation. In smartphone-edge pipelines, the same trade-off appears as perceived delay in user-facing outputs, where local computation can still yield several seconds of lag due to runtime and software constraints [25].

#### 2.2.2. Multimodal Alignment: Practical Strategies and What Can Go Wrong

Multimodal wearables add a third primitive: time alignment. Different sensors often run at different sampling rates and may have independent clocks, so the stream is not naturally “row-aligned” in the way a table is. A practical stream representation needs (i) a time base, (ii) a resampling policy, and (iii) a strategy for missing channels. Reviews of multimodal wearable sensing emphasise that preprocessing must handle both complex noise and coordination across modalities with different sampling rates and recording mechanisms [26]. In practice, three alignment patterns are common: (i) master clock alignment (one clock defines time and other streams are interpolated), (ii) timestamp alignment (each packet carries timestamps and streams are aligned at ingest), and (iii) signal-based alignment (a shared signal type allows cross-correlation to estimate delay).

For multi-device systems, the alignment problem is more explicit. Wang et al. developed Bluetooth Low Energy application-layer synchronisation plus data alignment for multi-channel wearable biosensors, showing that linear interpolation alignment reduced timing error compared with simpler packet-level adjustment, achieving sub-millisecond average alignment errors in their evaluation [27]. Xiao et al. addressed a complementary case where a common signal type is available across devices, using cross-correlation alignment and reporting improved synchronisation delay when signal quality filtering is applied [28]. These examples illustrate a practical point: alignment quality is coupled with signal quality, so SQI gating can improve synchronisation as well as downstream inference.

Alignment failures are common in the field and have predictable signatures. Packet loss or jitter can create time varying delays, causing modality “slip” that contaminates early-fusion representations. Boundary mixing can occur when partial windows are filled with stale samples after dropouts, which can bias short-window classifiers. Mulazzi’s real-time framework shows how link conditions and buffering affect throughput, partial windows, and decision latency, and reports that explicit visibility signals (mask channels) improved robustness when sensors dropped out [29]. The same work highlights the role of queue and back-pressure policies: noncritical streams may need to be dropped before the model path to preserve real-time behaviour [29]. For system developers, this suggests that alignment is not only a preprocessing step, but a runtime policy that must be stress tested under realistic packet loss and disconnection patterns.

#### 2.2.3. Feature and Update Rates as Response-Time Design

Finally, the stream structure must connect feature and update rates to clinically meaningful response times. Update rate is not simply “how fast the sensor samples”. It is the cadence at which the pipeline produces a usable output after buffering, alignment, and any smoothing across windows. Real systems show how these rates can differ by an order of magnitude. Smartphone-based edge analysis can run locally without cloud dependence but still present outputs with a small display delay because of software and runtime constraints [25]. At the other extreme, embedded classifiers can execute inference in the sub-second range on low-power hardware [24], and improved alignment can reduce median decision latency by avoiding boundary mixing in short windows [29]. For the remainder of this review, we therefore report stream design choices using three operational quantities: (i) the stride at which the model output is refreshed, (ii) the decision latency (time from physiological change to a stable output), and (iii) the availability policy (what the system does when channels are missing or low quality).

### 2.3. Labelling and Ground Truth

In wearable health monitoring, labels are rarely “attached” to the signal in a clean way. They are usually obtained through a second measurement process (for example, a reference device), a human decision (clinical adjudication), or a participant report. Each route introduces delay, noise, and missingness, which can dominate model performance if it is not made explicit in the study design and reporting. This is especially true for real-time systems, where the model consumes short windows but the clinical phenomenon may evolve over minutes to days.

#### 2.3.1. Where Labels Come From

A strong option is clinical adjudication against a reference standard, such as an ECG patch for atrial fibrillation (AF) detection. Poh et al. validated continuous, wrist-worn AF detection by comparing 15 min intervals against a 14-day ECG patch (Zio XT, iRhythm Technologies, Inc., San Francisco, CA, USA), which provides a clear, time-stamped reference for interval-level labels [30]. Even in this setting, the label stream is not perfect: a sizeable fraction of photoplethysmography (PPG) intervals may be marked as unanalysable, and performance estimates can shift sharply under worst-case assumptions about how those intervals relate to the reference labels [30]. These details matter for translational use because they affect alert burden, clinical review workload, and the interpretation of “negative” periods.

A second route is device-derived proxy labels. Sleep is a typical example: polysomnography (PSG) is treated as the clinical standard, but many studies rely on actigraphy outputs as a proxy when PSG is not available. Large-scale validation work shows that traditional actigraphy algorithms can achieve reasonable epoch-by-epoch accuracy against PSG in community cohorts, yet still struggle with agreement on derived sleep metrics such as wake after sleep onset, particularly in irregular sleep or sleep disorders [31]. More broadly, a scoping review of wearable sleep staging highlights that even PSG-based staging is not perfectly consistent across scorers, with reported inter-scorer agreement below full concordance, and it calls for consistent reporting and stronger validation practice for consumer-facing sleep technologies [32]. The practical takeaway is that a “ground truth” label may itself contain uncertainty, and the paper should state how that uncertainty was handled (or bounded).

A third route is self-report and ecological momentary assessment (EMA). Bell et al. used time-triggered and event-triggered EMA to confirm eating events as ground truth for smartwatch-based detection in the field [33]. EMA can reduce recall bias compared with retrospective diaries, but it still introduces compliance effects, timing jitter (responses are not instantaneous), and context-dependent missingness. For many applications, self-report should be treated as a noisy measurement with known failure modes, rather than as a perfect label.

A fourth route is weak labels and sparse clinical labels. This appears when outcomes are anchored to infrequent clinical events (for example, lab draws) while the wearable stream is continuous. Chung et al. make this issue concrete for PPG-based biomarker prediction: supervision becomes less reliable as the time gap between a signal segment and its corresponding lab value grows, and they propose weighting training examples by an explicit time gap decay [34]. The broader point for this review is that time is part of the label: when the label is delayed, the study must justify the supervision window and state how temporal distance was accounted for (discarding distant segments, down-weighting, or modelling uncertainty).

#### 2.3.2. Task-Specific Ground Truth Challenges

The right label depends on the clinical decision. For AF, PPG irregularity is not equivalent to a confirmed rhythm diagnosis; high-quality studies therefore ground evaluation in a reference ECG stream and report performance at a clinically interpretable granularity (for example, interval-level detection) [30]. For sleep, “sleep stage” implies a PSG definition, while actigraphy is closer to sleep–wake estimation; mixing these concepts without clear definitions can lead to over-claimed conclusions [31,32]. For human activity recognition, dense labels are expensive and may be unavailable in free-living settings; work that explores weak supervision or label-efficient strategies is partly motivated by this practical constraint [35]. Across these tasks, the core issue is that models are often trained on what is available, not what is clinically decisive; the review should highlight this mismatch and how it affects deployment claims.

#### 2.3.3. Reporting to Avoid Leakage and over Optimism

Because wearable streams are highly autocorrelated, small design choices can create silent leakage. Methodological surveys of digital biomarker work report recurring pitfalls such as participant-level mixing, overlapping time windows across train and test, and weak ground-truthing due to misaligned timestamps or recall-biased self-report [36]. These pitfalls are preventable if studies report label provenance and alignment with enough detail for reproducibility. For this review, we recommend that papers developing or evaluating wearable labels explicitly report the following:

- Label provenance:who or what produced the label (clinician adjudication, PSG scorer, reference device, EMA prompt, or lab system), and whether labels were reviewed or corrected [30,32,33].
- Time semantics: the label timestamp definition (onset, midpoint, and end), the epoch length, and the mapping from continuous streams to label intervals, including any tolerance window [30,31].
- Uncertainty and missingness: rates of unanalysable segments, non-response to EMA, and inter-scorer variability when relevant, plus how these were handled in evaluation [30,32,33].
- Split hygiene: subject-level splits as a default, and clear rules preventing overlap of highly correlated windows across splits, especially when using sliding windows or long recordings [36].
- Temporal distance controls: for sparse clinical labels, the distribution of time gaps and the chosen strategy (exclusion, weighting, or explicit modelling) to prevent stale supervision from dominating results [34].

Taken together, these practices shift the discussion from “label availability” to “label validity”. For translational bioengineering, that shift is essential: it clarifies what the system is truly learning, what it can safely claim, and where the remaining uncertainty sits when the model is moved from controlled studies to real-world monitoring.

## 3. Edge ML Methods for Streaming Biosignals

Edge deployment changes biosignal modelling from a purely predictive exercise into a joint systems-and-clinical design problem. The same model family can behave very differently once it must run continuously on a wearable or phone, under strict constraints on memory, energy, and latency. Recent embedded demonstrations show that clinically relevant signal understanding can be achieved on constrained hardware, but only when the pipeline is built with deployment in mind, including input segment design, quality control, and model compression (e.g., quantisation and pruning) [37,38]. At the same time, end-to-end edge health systems often require more than a classifier: they must handle secure communications, intermittent connectivity, and auditability, which motivates system-level architectures that combine edge inference with privacy-preserving collaboration and secure logging [39].

Within this review, we organise edge methods around three practical choices that recur across modalities and tasks: (i) representation (engineered features, learned representations, or hybrids), (ii) model family (convolutional, recurrent, attention-based, or combinations), and (iii) adaptation strategy (compression, personalisation, and pretraining). These choices interact with the streaming structure introduced in Section 2, because window length, overlap, and update rate effectively set the information available to the model and the compute budget available to the device at each decision point.

### 3.1. Representations and Model Families

#### 3.1.1. A Decision-Oriented View: Choose the Model to Match the Stream and the Device

Section 2 showed that streaming design fixes both the information available to the model (window length, overlap, and update rate) and the compute budget per decision. A practical way to choose representations and model families is therefore to start from the deployment requirement.

*If you need short windows with low latency and predictable energy*, favour feature-based pipelines or compact convolutional models (CNN/TCN), which typically have stable runtimes and compress well. Embedded demonstrations show that quantised CNN inference on microcontrollers is feasible for ECG tasks, with explicit quality screening to avoid wasting compute on unusable segments [37]. In low-power ventricular fibrillation detection, post-training quantisation reduced model footprint and cut latency and energy substantially (for example, flash 40 kB to 20 kB and latency about 4.8 s to about 0.6 s on the target microcontroller, with large energy reduction), illustrating that compression can dominate end-to-end feasibility [40]. These numbers are workload and platform specific, but they provide realistic orders of magnitude for what “edge feasible” can mean in practice.

*If you need longer temporal context under edge constraints*, hybrid designs are often the best compromise. Convolutions capture local morphology cheaply, while a lightweight recurrent or attention component integrates broader context when needed. DCETEN is a representative example that combines a 1D CNN front-end with a Transformer encoder and then improves edge suitability using depthwise separable convolution, channel attention, and pruning [38]. The general principle is that the expensive mechanism should be applied after the sequence has been shortened or compressed by earlier layers, otherwise the edge cost scales unfavourably.

*If you have limited adjudicated labels, or need transfer across devices and populations*, pretrained encoders with small task heads can be more reliable than training from scratch, provided the encoder can be reused without repeated on-device updates. Surveys of biosignal foundation models emphasise that heterogeneity (sampling rates, channel configurations, and missing channels) is a central obstacle, and they position self-supervised pretraining as a route to more transferable representations [41]. ECG-FM provides an open ECG encoder intended for transfer, particularly when labels are scarce [42], and related work explores single-lead wearable ECG representation learning via proxy supervision from richer clinical sources [43]. In edge settings, the most practical pattern is to keep the encoder fixed and adapt only a small head or calibration layer, which reduces both compute cost and governance risk.

#### 3.1.2. Classical Feature Pipelines and Why They Persist at the Edge

Feature-based pipelines remain common because they separate signal understanding into transparent steps: denoising, beat or event detection, feature extraction, and then a compact classifier. For ECG, this often includes morphology summaries and rhythm features, including RR-interval-derived descriptors that remain attractive on-device due to their low dimensionality and clear physiological meaning [38]. In embedded biometric ECG, frequency domain representations can also be competitive in selected subjects, and simple classifiers may remain viable when the representation is stable, which highlights a recurring theme for edge design: representation quality can sometimes compensate for model simplicity [37]. Broadly, this is consistent with the wider wearable time-series literature, where classical methods are valued for interpretability and predictable resource use [44].

#### 3.1.3. Learned Representations from Raw Streams

Learned representations dominate many recent benchmarks because they exploit subtle morphology and temporal contexts that are difficult to hand code [45,46]. Convolutional models are often the first choice for single-channel streams because they are efficient and have a controllable receptive field. A quantised CNN deployed on a microcontroller for ECG biometrics demonstrates a practical edge pipeline that combines short fixed-length segments with quality screening [37]. For longer context, lightweight hybrids are increasingly used to balance local extraction and broader temporal integration. DCETEN provides one example, pairing CNN feature extraction with a Transformer encoder and adding pruning-oriented design choices [38]. In practice, these hybrids are most defensible when the task genuinely needs context beyond a short window (for example, rhythm patterns rather than isolated beats) and when their edge costs are reported transparently.

Table 2 summarises the main model families used for streaming biosignals at the edge, highlighting typical strengths, limitations, edge suitability, and common modalities or tasks.

#### 3.1.4. Which Families Dominate Which Tasks and Modalities, and What This Implies for Deployment

Across ECG beat and rhythm classification, recent reviews describe a shift from manual feature engineering towards architectures with automated feature extraction, including CNNs, recurrent models, and hybrids with attention [46]. For many ECG tasks, compact CNN or TCN variants remain the most common edge-friendly choice because they are compressible and can meet fixed update-rate constraints. For inertial sensing (activity recognition and gait-related tasks), CNN and LSTM-family models remain widely used because they map naturally onto short sliding windows and capture temporal structure without heavy hand-engineering [47]. The deployment implication is straightforward: model choice must reflect the windowing and update cadence, because a model that is accurate but cannot refresh outputs at the required rate, or that consumes too much energy for continuous operation, will fail in practice.

For this reason, it is useful to report representative on-target ranges rather than a single best-case number. Embedded studies show that microcontroller inference can range from sub-second to multi-second per decision depending on model size and runtime path, and that compression can change latency and energy by multiples rather than marginal percentages [40]. Section 4 and Table 3 consolidate these deployment levers and their typical risks.

#### 3.1.5. Self-Supervised Pretraining and When It Helps in Low-Label Regimes

Self-supervised pretraining reduces dependence on large labelled datasets, which are costly for clinical-grade ground truth. Surveys group the field into: training large encoders from biosignal corpora, adapting general time-series models to biomedical signals, and leveraging language model tooling via signal-to-token or embedding interfaces [41]. These surveys also highlight obstacles that matter in wearables, such as differing sampling rates, channel configurations, and missing channels at deployment [41]. Concrete examples include open ECG encoders trained at scale, such as ECG-FM [42], and single-lead wearable ECG representation learning via proxy supervision from richer clinical sources [43]. For edge translation, pretraining is most useful when (i) the downstream task has limited adjudicated labels, (ii) generalisation across devices or populations is required, and (iii) the pipeline can reuse a fixed encoder with a small head that fits the target latency and memory budget. When labels are abundant and task-specific, the marginal benefit is often smaller, and evaluation should confirm that pretraining improves reliability and not only headline accuracy.

### 3.2. Personalisation and Robustness

Wearable biosignals vary across people and also change within the same person over time. These two sources of variation can undermine a single “one-size” model, particularly in real-time edge settings where signals are short, noisy, and recorded in changing contexts. Recent work therefore treats personalisation as a core design goal, not an afterthought. For ventricular arrhythmia detection on single-lead ECG, Sun et al. explicitly target both inter-subject diversity and intra-subject variability by combining a meta-learning pretraining stage with a staged adaptation procedure, so the model can adapt with very limited per-person data [50]. In distributed settings, Jia et al. make a similar point in a federated framework, noting that a global model can be skewed by subject heterogeneity and that naïve local fine-tuning may overfit or drift when the individual’s future data differ from their fine-tuning data [51].

#### 3.2.1. Personalisation Strategies and the Cost of Calibration

Personalisation can be implemented at several levels. A simple but important layer is calibration and normalisation to correct device-specific differences. For example, when adapting a 12-lead ECG model to single-lead smartwatch ECGs in heart failure patients, Hempel et al. report that the smartwatch recordings were unitless and required an amplitude calibration factor derived from simultaneous recordings, which helped bridge differences between clinical and consumer hardware [52]. This illustrates a practical but underreported issue: calibration has a user burden. It may require an additional supervised measurement (or a paired reference device), extra clinic time, or repeated set-up steps, and these costs should be stated alongside performance.

Beyond signal calibration, model-level adaptation ranges from light fine-tuning to more structured approaches. Sun et al. show that rapid subject-level adaptation is feasible with very few labelled beats per class, and motivate meta-initialisation plus an additional pre-fine-tuning step to improve adaptation under intra-subject variability [50]. For continuous blood pressure estimation, Liu et al. combine multi-view wearable signals with transfer learning and a personalised fine-tuning strategy that adjusts selected layers while incorporating individual information, then use distillation to support lightweight deployment [53]. In federated settings, PMFed adds a domain-similarity guided aggregation scheme and an adaptive personalisation mechanism that selects personalisation parameters and fine-tuning rates based on subject similarity, aiming to preserve cross-subject representations while improving performance for unseen individuals [51]. From a deployment standpoint, the most robust pattern is often to keep the bulk of the representation fixed and adapt only a small head or calibration layer, because this limits compute cost and reduces the chance of destabilising the model.

#### 3.2.2. On-Device Adaptation Constraints: Privacy, Drift, and Catastrophic Forgetting

On-device adaptation is appealing because it can reduce reliance on data transfer and can tailor models to individual baselines. It also introduces hard constraints that are often glossed over. First, adaptation consumes energy and competes with real-time latency budgets. Second, repeated fine-tuning can create drift, particularly when the individual’s recent data are not representative of their future data, or when adaptation is triggered during periods of poor signal quality [51]. Third, continual updates can cause catastrophic forgetting, where the model improves on the current context but loses performance on previously seen contexts or activities. Approaches that stabilise updates through regularisation, replay, or distillation are therefore important when adaptation is performed over time (rather than as a one-off calibration). Privacy is also a practical constraint: even when raw signals remain on-device, telemetry, gradients, or update statistics can still leak sensitive information if they are transmitted without safeguards. For these reasons, personalisation should be treated as a governed process with explicit rules: what is updated, how often, under what quality conditions, and how rollback is handled when performance degrades.

#### 3.2.3. Robustness to Artefacts and Missingness

Robustness methods can be grouped into quality gating, augmentation, adaptation, and missing-data handling, each with different on-device costs. Personalisation only helps if the model remains stable under noisy, incomplete, and artefact-prone streams. Consumer wearables routinely record with more motion artefact and muscle noise than clinical systems. In smartwatch ECG classification, Hempel et al. observe that correctly classified windows tended to have higher signal-to-noise ratio than misclassified ones, but also report that simple median filtering handled much of the noise and that excluding low-quality segments did not necessarily improve performance [52]. Their results suggest that aggregation choices matter in streaming settings: a maximum-based aggregation over short segments improved sensitivity and F1 compared with median aggregation, implying that brief “outlier” windows can carry decisive rhythm cues [52]. For PPG, a separate line of work focuses on identifying when the signal is usable before computing downstream endpoints such as heart rate. For instance, methods that explicitly separate motion artefact detection from usability detection can discriminate usable from non-usable segments for heart-rate estimation [54]. At a broader systems level, reviews underline that motion artefacts, power-line interference, false detections, and dry-electrode issues are recurring barriers, and that artefact suppression and multi-sensor fusion can improve reliability [16].

#### 3.2.4. Domain Shift, Federated Personalisation, and Calibration Monitoring

Domain shift in wearables is multi-factorial. It can be caused by device models and sensor front-ends, lead configurations, wear location, and the activity context of recording. The 12-lead to 1-lead adaptation study illustrates this: performance drops were rhythm dependent, and the relation between noise and model confidence differed by rhythm class, reinforcing the need to report per-task behaviour rather than only pooled metrics [52]. For PPG heart rate estimation, Kim et al. frame cross-subject generalisation as a domain adaptation problem and propose multi-source partial domain adaptation using only unlabeled target-subject PPG, explicitly handling cases where the target heart rate range only partially overlaps the source ranges [55].

Federated learning can support privacy-preserving personalisation by keeping raw data local while updating shared parameters, but it does not remove the need for monitoring. In practice, deployment should track at least two signals of instability: changes in input quality distributions (for example, unusable-window rates) and changes in calibration (for example, drift in calibration curves or summary calibration error) as devices, firmware, and user behaviour evolve. Evaluation should therefore include subject-level dispersion, tests on unseen subjects and devices, and reporting that separates calibration, adaptation, and final evaluation [51]. In this review, we treat “good” robustness evidence as reporting that makes shift visible, such as subject-wise and device-aware splits, per-subject or per-device breakdowns, explicit handling of low-quality or missing segments, and clear statements of adaptation cost (including calibration burden) and update governance.

### 3.3. Multimodal Fusion on Device

Multimodal fusion is attractive in wearables because different sensors fail in different ways. Motion can corrupt PPG while an accelerometer remains informative, and electrode issues can weaken ECG while a secondary channel may still track rhythm-related changes. In practice, fusion is most useful when it either (i) increases coverage across activity contexts, or (ii) reduces false alarms by allowing the model to cross-check evidence across modalities.

#### 3.3.1. Fusion Designs and Edge Costs

Fusion designs are commonly grouped into early, late, and hybrid strategies. Early fusion combines modalities before the main model, for example by concatenating aligned windows or by summing feature maps. This can capture cross-modal interactions directly, but it pushes costs onto the edge because it increases input width, requires tight time alignment, and can raise memory pressure for buffering. Late fusion processes each modality with its own lightweight encoder and combines decisions or logits. This is often easier to schedule on device because each encoder can run only when its input is present and of acceptable quality, but total compute can rise if multiple encoders run at every update. Hybrid fusion combines both, for example using modality-specific encoders followed by a shared fusion block, and is often the most practical compromise when compute budgets are tight and modality quality varies rapidly [56].

#### 3.3.2. When Fusion Improves Results on Device

Empirical results increasingly show that fusion is not only a modelling choice but also a systems choice. In BrainFuseNet, seizure detection improves when EEG is fused with wrist signals (PPG and accelerometry), and the authors report that early-fusion variants trade sensitivity and specificity differently (for example, concatenation versus weighted summation), highlighting that fusion rules matter as much as the backbone [57]. Importantly for edge deployment, BrainFuseNet was implemented on a GAP9-class platform with millisecond-scale inference times and very low energy per inference, illustrating that carefully designed fusion models can remain feasible under wearable power limits [57]. This is a useful pattern for other clinical monitoring tasks where one modality is strong but fragile and the secondary modality is weak but robust.

#### 3.3.3. Handling Missing Modalities and Variable Quality

Real deployments must assume that at least one channel will be intermittently missing or unreliable. A practical approach is to make fusion quality aware. MLFusion is a clear example: it treats signal quality indicators (SQIs) as explicit inputs alongside ECG and PPG streams, and evaluates robustness under simulated noise, which matches the reality that wearable signals can be intermittently corrupted by motion, contact changes, or interference [58]. In this setting, a sensible design is to combine (i) SQI gating at the input level (drop or downweight low-quality windows), with (ii) a fusion rule that degrades gracefully when one modality is absent (confidence-weighted fusion, modality dropout during training, or mixture-of-experts routing). Recent work on multimodal physiological foundation models also supports this direction: cross-modal reconstruction objectives and modality dropout can improve downstream performance, while some late-fusion contrastive pretraining strategies appear less effective across diverse tasks [59]. Architectures that explicitly model arbitrary missingness using mixture-of-experts style routing and sparse cross-modal attention are also being explored to avoid hard assumptions about which modality is “primary” [60].

#### 3.3.4. Hardware and Synchronised Acquisition as Enablers

Fusion performance on paper can collapse if the device cannot acquire, synchronise, and buffer streams reliably. BioGAP-Ultra is a useful reference point because it emphasises modular, synchronised multimodal acquisition (EEG/EMG/ECG/PPG and inertial signals), paired with on-device processing and practical power characterisation across form factors (for example, headband, sleeve, and chestband configurations) [61]. From a deployment standpoint, this underlines a key lesson: multimodal fusion is only clinically useful when timing alignment, buffering during wireless dropouts, and end-to-end power management are addressed together with the model design [61].

#### 3.3.5. When Fusion Is Not Worth It

**Figure 1:**
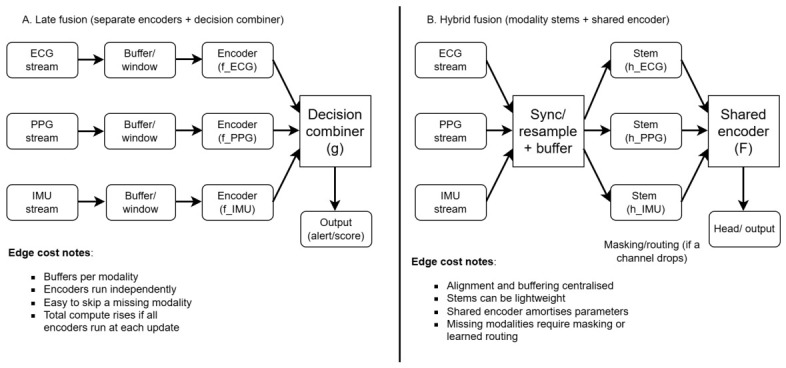
Late fusion versus hybrid on-device fusion for wearable biosignals. (**A**) Late fusion runs separate modality encoders and combines outputs, which simplifies skipping a missing modality but can increase total compute when all encoders run at each update. (**B**) Hybrid fusion centralises synchronisation and buffering, uses lightweight modality stems, and shares a common encoder, but requires masking or routing when a channel drops.

Fusion adds complexity that can be hard to justify if a single modality already meets the clinical requirement. If the downstream task tolerates occasional missingness (for example, trend monitoring rather than instant alarms), or if one sensor dominates performance and the second modality rarely changes decisions, a simpler unimodal pipeline may be preferable. In this review, we therefore treat fusion as a decision guided by (i) complementarity of failure modes, (ii) the expected operating context (ambulatory and motion-rich versus sedentary), and (iii) whether the device can support the added buffering, synchronisation, and compute costs without shortening battery life beyond acceptable limits. As shown in Figure 1, late fusion and hybrid fusion differ in where buffering and alignment occur and in how gracefully the pipeline can handle a missing modality.

## 4. Deployment Engineering and Resource-Aware Optimisation

Deploying streaming biosignal models in practice is often limited less by headline accuracy and more by whether the model can run within a tight budget for latency, memory, and energy. Benchmarking work on low-power wearables shows that energy can be dominated by different phases (idle, acquisition, and processing), and that no single platform is best across workloads, which makes hardware-aware reporting essential [62]. In this setting, compression and acceleration are not optional clean-up steps. They are the core engineering levers that decide whether a model can run continuously, update frequently enough to be clinically useful, and remain stable under real-world noise.

### 4.1. Compression and Acceleration

Three levers recur across edge biosignal deployments: quantisation, pruning, and distillation. Quantisation typically provides the most immediate benefit because it reduces both model size and the cost of arithmetic, especially when the runtime supports integer kernels. A clear illustration is ventricular fibrillation detection where post-training quantisation reduced flash footprint (40 kB to 20 kB), cut latency on a low-resource microcontroller (about 4.8 s to about 0.6 s), and reduced energy by roughly 7.85× [40]. Importantly, this study also highlights a practical failure mode: some “hybrid” quantisation paths are not supported on certain microcontroller runtimes, so the deployment route must be reported along with the model [40]. In a separate edge ECG anomaly-detection pipeline, pruning and quantisation were combined to lower power and improve throughput, but with non-trivial trade-offs in accuracy depending on which optimisation was applied [48]. Taken together, these results support a simple rule for biosignals: apply quantisation early, then use pruning to target the residual bottlenecks and verify that the full signal-processing chain remains stable.

Pruning is attractive because biosignal models often contain redundancy, yet pruning does not always translate into end-to-end speedups unless the sparsity pattern matches what the compiler or accelerator can exploit. Structured pruning (channel or filter removal) is more likely to reduce latency, while unstructured pruning mainly helps storage and can require specialised kernels. In the ECG anomaly detection example, pruning maintained power consumption while reducing accuracy relative to the original model, which underlines the need to report where pruning is applied and which layers are protected [48]. For streaming inference, it is also worth checking whether pruning amplifies sensitivity to artefacts, because small distortions can cascade through short windows.

Distillation is often the most practical route when the best-performing model family is too heavy for a wearable. In PPG heart rate estimation, multiple distillation strategies were compared and distillation consistently improved small models over training from scratch; decoupled distillation performed best in that study [49]. The same line of work reports that compressing a large model to a very small student can yield large reductions in inference time and memory, with a measurable but sometimes acceptable loss in error metrics [49]. For wearable biosignals, the key advantage is that distillation can preserve task performance while allowing a simpler student architecture that is easier to quantise and to run with predictable latency.

Across all three levers, the review literature repeatedly shows that deployment claims are hard to interpret without consistent reporting. At minimum, studies should report: model format and runtime (e.g., LiteRT/TFLite and vendor SDK), quantisation type (dynamic range, int8, full-int8, or quantisation-aware training), calibration or representative dataset strategy, pruning schedule and sparsity structure, and the exact measurement protocol for latency and energy (sampling rate, window size, overlap, batching, clock settings, and power measurement method). For instance, ECG anomaly-detection work explicitly reports model size, compression ratio, inference time, throughput, and device-side resource metrics such as CPU and memory utilisation [48]. Where feasible, authors should include reproducible deployment configurations (toolchain versions, compiler flags, and scripts) as supplementary material, because small differences in runtime kernels can change the latency and energy profile materially.

### 4.2. Runtime Constraints and Benchmarking

Compression results are only meaningful if the deployed system meets the runtime constraints of continuous sensing. For streaming biosignal models, benchmarking should therefore capture (i) on-target accuracy (after porting and quantisation), (ii) latency per window and end-to-end decision latency (including buffering and preprocessing), (iii) peak RAM (activations, buffers, intermediate tensors, and runtime overhead), (iv) flash or storage footprint (model weights plus runtime libraries), and (v) energy under a clearly stated duty cycle. Practical benchmarking toolchains such as MLino Bench help make these measurements repeatable by streamlining training, porting, flashing, and on-device evaluation, while reporting core metrics such as on-target accuracy, classification time, and model size [63]. At the community level, MLPerf Tiny offers a shared reference point for reporting latency, energy, and accuracy on resource-constrained platforms [64].

#### 4.2.1. Measurement Protocols

Benchmark protocols should reflect the intended deployment conditions rather than idealised lab runs. At minimum, authors should fix realistic sampling rates and window sizes, include any overlap and buffering, run enough inferences to reach stable timing, and report distributions (e.g., median and tail latency) rather than a single number. Energy measurement needs particular care. A common failure mode is to include non-inference phases (data movement, preprocessing, and idle loops) inside the measurement window, which can underestimate true inference costs and make comparisons unfair. Recent work has shown that more selective per-inference measurement, with explicit synchronisation between current sampling and inference timing, can materially change reported energy and reduce measurement uncertainty compared with MLCommons-style counting methods [65]. In practice, reporting energy per inference (or per decision) is often more informative than instantaneous power, because modest power differences can be outweighed by large changes in execution time [65].

#### 4.2.2. Acceleration, Utilisation, and What to Report

Hardware accelerators can shift the feasible design space, but their benefits depend strongly on utilisation and model complexity. On a Cortex-M55 + Ethos-U55 platform, NPU offload delivered large latency and per-inference net energy reductions for moderate-to-large models, while very small models could regress due to orchestration overhead [66]. This work also highlights that per-inference net energy (idle-subtracted, inference-synchronised) is a meaningful metric for embedded decisions, and that memory hierarchy and data movement remain limiting factors when activation buffers are large [66]. For biosignal deployments, authors should therefore report not only whether an accelerator is present, but also the toolchain, supported operators, and any observed utilisation or bottlenecks that explain performance.

#### 4.2.3. Thermals and Sustained Operation

Wearables are constrained by user comfort and safety, so benchmarks should consider sustained runs and thermal behaviour, not only short bursts. Thermal footprints vary over time and across device locations; single-value temperature reporting is often misleading. Prior work proposes reporting saturation temperature and saturation rate per component, and emphasises spatial measurement (e.g., CPU, accelerator, and SRAM) for skin-contact devices [67]. Practical factors such as supply voltage and enclosure can materially change thermal behaviour and should be documented, because trapped heat can drive temperatures beyond typical comfort limits during extended operation [67].

#### 4.2.4. Minimum Edge Cost Reporting Schema

To make benchmarking claims comparable and reproducible, we recommend reporting edge costs using a fixed schema. A simple way is to include the following “Edge Cost Card” in each paper.**Edge Cost Card (minimum reporting). Task + stream:** modality(ies); sampling rate(s); window length; stride/overlap; buffering policy; output update rate. **Edge tier:** device-only/device+phone/edge-assisted; compute placement (preprocessing, model, postprocessing). **Hardware:** board/SoC; CPU/NPU; clock; RAM; flash; supply voltage; accelerator delegate (if any). **Software:** runtime (version); compiler and optimisation flags; quantisation type (PTQ/QAT, int8/full-int8); operator fallbacks. **Latency:** per-window model inference time preprocessing (median, p95); end-to-end decision latency including buffering + preprocessing (median, p95); number of runs. **Memory:** peak RAM (buffers + activations + runtime overhead); model footprint on flash/storage (weights + libraries). **Energy:** energy per inference (or per decision); measurement instrument and trigger; idle subtraction rule; uncertainty if available. **Duty cycle:** sensing versus processing versus radio schedule; assumed usage; battery-life estimate method. **Thermals:** ambient; enclosure state; run duration; steady-state temperature (or saturation rate); throttling observed (yes/no). **Reproducibility:** code and configuration files; model version/hash; dataset split IDs; exact benchmark script.

### 4.3. Edge Architectures

**Figure 2:**
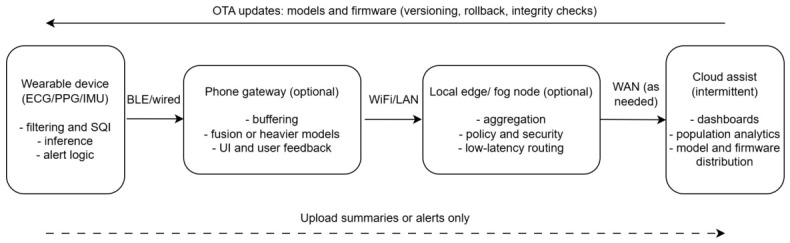
Tiered edge architectures for wearable biosignals. The wearable tier supports local pre-processing and inference; the phone and local edge/fog tiers (optional) provide buffering and heavier computation; cloud assist is used intermittently for dashboards and lifecycle management. Arrows indicate typical dataflow and the update pathway back to devices.

Edge deployment is not a single design choice, but a family of patterns that decide (i) where inference runs, (ii) what data leave the body, and (iii) how the system remains maintainable over months and years. In wearable biosignal systems, these decisions shape latency, privacy exposure, and robustness under intermittent connectivity. We therefore distinguish three practical architectures: device-only, device-plus-phone gateway, and intermittent cloud assist (Figure 2).

#### 4.3.1. Device-Only (Wearable-Native Inference)

In device-only designs, filtering, signal-quality checks, and the primary inference step run on the wearable itself, and the radio is used sparingly (for example, to transmit alerts or short summaries). This pattern is attractive for time-critical detection (such as fall events) and for privacy by design, because raw waveforms do not need to be streamed continuously. The trade-off is that on-device resources constrain buffering, model size, and post-processing, and debugging can be harder once devices are in the field. For long-lived deployments, device-only systems still need a safe update pathway (see below), otherwise performance and security can drift as conditions, devices, and threats change.

#### 4.3.2. Device + Phone Gateway (Phone-Edge)

A common compromise is to use the phone as a gateway and secondary compute tier: the wearable handles sensing and lightweight pre-processing, while the phone provides buffering, richer user feedback, and room for heavier models or late-stage fusion. This tiering is aligned with mHealth practice, where wearables are often treated as sub-devices that rely on a smartphone for interaction, connectivity, and broader sensing and analytics [68]. In chronic disease monitoring concepts, local processing is also motivated by reduced latency and reduced exposure of sensitive streams, while still enabling personalised feedback [69]. The main failure modes here are practical: Bluetooth congestion, background OS throttling, app lifecycle constraints, and gaps when the phone is absent or uncharged. A robust implementation therefore treats the phone tier as optional, and keeps a minimal safe behaviour on the wearable for critical alarms.

#### 4.3.3. Intermittent Cloud Assist and Local Edge or Fog Tiers

Cloud assist is best treated as intermittent and selective: it is used for clinician dashboards, longitudinal summaries, population analytics, and fleet management rather than as the only inference location. A local edge or fog node (home hub, ward gateway, or nearby server) can further reduce reliance on wide-area links by aggregating streams, enforcing security and routing policies, and performing time-sensitive computations near the data source [70]. Reported hybrid fog-edge patterns emphasise reduced end-to-end latency and bandwidth through local processing and selective transmission, with security monitoring split across tiers [70]. For wearable biosignals, the practical implication is that raw, high-rate streams need not be uploaded continuously; instead, edge tiers can trigger uploads when confidence is low, when events occur, or when retrospective review is required.

#### 4.3.4. Update Pathways and Operational Safety (OTA, Rollback, and Patching Reality)

Regardless of where inference runs, clinical-grade deployments must plan for lifecycle management. Evidence from IoT update architectures highlights why: reliable over-the-air updating benefits from explicit version control, branch separation (testing versus production), protocol diversity, integrity checks, and rollback-capable partitions to prevent device bricking under packet loss or partial transfers [71]. This is not a “nice to have” in healthcare environments. Studies of patching connected medical devices describe organisational constraints, including heterogeneous inventories, operational disruption, and the scaling limits of manual technician-based updates [72]. For a wearable edge pipeline, the minimum safe posture is: (i) signed artefacts, (ii) integrity verification before activation, (iii) rollback on failure, and (iv) staged rollouts with monitoring. These practices also align with regulatory expectations that cybersecurity is managed throughout the device lifecycle [73].

#### 4.3.5. Regulatory and Clinical Integration (Lifecycle, Risk Management, and Post-Market Surveillance)

If a wearable edge system is intended to influence care, its architecture must support lifecycle governance, not only initial model performance. From a regulatory perspective, expectations increasingly emphasise total product lifecycle controls, including cybersecurity-by-design and documented update pathways (versioning, integrity checks, and rollback) [74,75]. In parallel, the FDA has explicitly highlighted the need for approaches to measure and evaluate real-world performance of AI-enabled medical devices, including identifying and managing performance drift over time [76]. Practically, this means that design choices in Figure 2 should enable traceable model and firmware versions, logging of key operating signals (e.g., unusable-window rates, alert rates, and calibration drift), and a defined post-market surveillance plan with triggers for investigation, rollback, or revalidation. Organisational evidence on connected medical device patching also shows that updates carry workflow and inventory burdens, reinforcing the need for staged releases and clear ownership rather than ad hoc field changes [72].

#### 4.3.6. Which Architecture Fits Which Use Case

As a rule of thumb: (i) fall detection and safety alarms favour device-only inference with opportunistic phone or cloud reporting; (ii) screening tasks (for example, AF screening) often work well with device-plus-phone, where the phone enables better buffering, user prompts, and escalation workflows; and (iii) longitudinal management (chronic disease monitoring) benefits from a tiered approach where on-body decisions are fast and local, while cloud assist supports trends, clinician review, and fleet updates [68,69]. In all cases, architecture should be reported as a reproducible dataflow: what runs where, what is transmitted, at what rate, and under which triggers (Figure 2).

## 5. Applications and Translational Reliability

Edge ML for wearables is ultimately judged by whether it supports safe and useful decisions outside controlled laboratory conditions. In real deployments, accuracy alone is not enough. Systems must cope with low-quality segments and unusable windows, user adherence and wear compliance, false-alert burden, and shifting operating conditions, while fitting into clinical workflows and update cycles. This section summarises the main application areas and provides a balanced appraisal of deployment hazards, validation depth, and reliability practices that most often limit translation.

### 5.1. Use-Case Landscape

To keep the landscape tractable, we group applications into four buckets that align with typical signals, outputs, and deployment constraints. Across all buckets, translation is often limited less by headline accuracy and more by operational realities: false-alert burden, adherence and wear compliance, quality-gating rates (unusable windows), and how outputs fit into clinical workflows.

#### 5.1.1. Cardio: Rhythm Screening and Longitudinal Arrhythmia Monitoring

The most mature wearable use cases involve rhythm assessment, especially atrial fibrillation (AF) screening and monitoring. Typical outputs include irregular-rhythm notifications, episode detection, and AF burden estimates over days to weeks [77,78]. In practice, cardio pipelines frequently rely on quality-gated analysis windows because motion and contact artefacts can dominate free-living PPG, leading to large fractions of unusable segments [77]. This creates a direct deployment constraint: high unusable-window rates reduce effective monitoring coverage and can delay detection, particularly if the model requires several consecutive usable windows before triggering an alert. Several systems therefore pair continuous PPG screening with confirmatory spot-check ECG, which can reduce false positives and give clinicians a familiar strip for review [77]. Key failure modes include (i) false positives driven by ectopy or non-AF arrhythmias, (ii) demographic and device variability (including sensor differences and skin tone-related optical effects), and (iii) alert fatigue or workflow burden when inconclusive traces require clinician review [78]. For bedside translation, studies should therefore report not only discrimination, but also false alerts per day (or per monitored hour), the rate of inconclusive or unusable segments, and the operational pathway for confirmatory testing and review.

#### 5.1.2. BP and Haemodynamics: Cuffless Blood Pressure and Vascular Proxies

Cuffless BP aims to move beyond occasional readings towards frequent, low-burden monitoring. Common outputs include SBP/DBP estimates, trends, and variability summaries, often derived from PPG-based features and timing proxies such as PAT/PTT or pulse-wave analysis [79,80]. The dominant translational challenge is that these measurements are typically inferred rather than directly measured, so calibration, drift, and physiological confounding (heart rate, vascular tone, posture, temperature, and stress) can degrade accuracy in daily life [79]. This makes adherence and calibration burden central: many approaches require initial calibration against a cuff measurement and may require repeat calibration over time, which can limit usability and long-term uptake. Edge deployment also introduces a trade-off between artefact suppression (more filtering and quality control) and timely updates; conservative quality gating may improve accuracy but reduce coverage during activity. Validation should therefore include dynamic conditions and diverse populations, report calibration procedures explicitly, and quantify how often the system produces a valid estimate in free-living use [79,80].

#### 5.1.3. Sleep and Respiratory Monitoring: Staging, Apnoea Proxies, and Nightly Summaries

Sleep staging is a high-impact use case but remains methodologically difficult because the gold standard (polysomnography) is intrusive and manual scoring has non-trivial inter-scorer variability [81]. Wearables often report (i) summary measures (total sleep time, wake after sleep onset, and sleep efficiency) and (ii) epoch-by-epoch stage labels, with the latter being more informative for classifier evaluation [81]. Recent work and reviews emphasise that multi-sensor combinations (accelerometry with PPG and temperature) can help, but comparisons are complicated when algorithms are proprietary, epoch definitions vary, or time synchronisation is imperfect [81]. Practical failure modes include label noise, temporal misalignment between sensors and reference, and limited access to raw epochs for independent validation. In deployment, adherence and comfort matter: devices may be removed overnight, lose contact, or record with lower optical quality, leading to missing epochs and biased sleep summaries. For bedside credibility, it is useful to report the missing-epoch rate, any quality-gating criteria, and how often valid nightly summaries are produced under free-living conditions, rather than reporting only best-case epoch accuracy.

#### 5.1.4. Movement, Function, and Stress: Falls, Frailty, and Psychophysiology

IMU-driven applications (fall detection, activity recognition, and gait-based functional assessment) are well suited to edge operation because inertial sensing can be processed locally with modest compute. Outputs range from discrete events (fall detected) to longitudinal functional indicators (gait variability and frailty risk) [82,83]. A key translational advantage of on-device inference is reduced dependence on continuous connectivity and reduced raw data handling, which can improve feasibility for multi-day monitoring at high sampling rates [83]. However, critical appraisal is needed. Fall detection has clear event definitions but remains sensitive to wear location, user behaviour, and gateway assumptions (for example, reliance on a phone nearby) [82]. False alarms can impose substantial caregiver or clinical burden, while missed events carry obvious safety risk, so reporting should include false alarms per day and performance under realistic activity contexts. Frailty and functional assessment are promising but often limited by small cohorts, subject-specific effects, and the need for stronger external validation before routine clinical use [83]. Stress and related wellness endpoints are even more challenging because ground truth is context dependent and easily confounded by physical activity, temperature, and individual baselines; robust evaluation often requires multimodal measurement and carefully defined protocols [84]. In practice, user burden (wearing multiple sensors and responding to prompts) and missing self-reports can dominate data quality, so studies should report adherence rates and the extent of missing labels.

#### 5.1.5. Mature Versus Emerging Use Cases

Within this review, we consider a use case relatively mature when (i) it has repeated validation against an accepted reference standard (e.g., Holter for AF or PSG for sleep), (ii) it reports failure modes and data exclusion explicitly (including unusable-window rates and missingness), and (iii) it provides evidence on adherence, usability, and operational burden (for example, false-alert rates and clinician review workload). Under these criteria, AF detection and monitoring is comparatively mature, while cuffless BP, sleep staging beyond coarse summaries, and stress inference remain emerging. Gait-based frailty assessment sits between these extremes: it is clinically compelling, but still needs broader cohort evidence, deployment studies, and clearer reporting of adherence and workflow impact. Table 4 provides a consolidated comparison across use cases, signals, model families, edge tiers, and deployment pitfalls.

### 5.2. Validation That Translates

**Figure 3:**
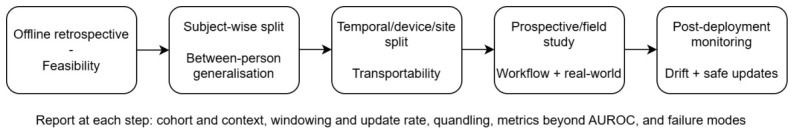
Validation ladder for wearable edge monitoring. Each stage answers a different question, from offline feasibility to real-world workflow performance and post-deployment stability.

Wearable edge systems are evaluated on streams, but they are used in settings where behaviour, signal quality, and device handling change from day to day. Validation should therefore be framed as evidence that the system will remain reliable under the conditions it will actually face, rather than as a single offline performance number. Figure 3 summarises a practical validation ladder for moving from retrospective analysis to field use and post-deployment surveillance.

#### 5.2.1. Validation Designs and What Each Proves

*Subject-wise splits* are the default for most wearable tasks because they test between-person generalisation. They reduce the risk that a model simply learns person-specific signatures that do not carry over to new users. In activity and gait monitoring, leave-one-subject-out or identity-disjoint splits are commonly used to avoid leakage from highly correlated windows [83]. For problems where signals evolve over time, *temporal splits* provide a more realistic stress test by training on earlier periods and testing on later periods, which exposes drift due to behaviour change, sensor ageing, or firmware updates. Where multiple devices or sites are involved, *device or site splits* assess transportability across hardware front-ends and data collection routines. This matters in practice because sampling rates, filtering, and contact conditions can differ by device even when the nominal modality is the same [20]. Finally, *prospective or field studies* establish whether the full pipeline holds in free-living use, including adherence, motion artefact, and gateway availability. Field evidence often depends on realistic ground-truth collection, such as ecological momentary assessment for event confirmation, or reference instruments used in parallel [33,77].

External validation should be treated as a minimum expectation for mature use cases. For example, embedded AF screening work reports performance on an independent long-term AF database after training on a separate source, which provides a clearer picture of generalisation than a single internal split [24]. In functional assessment, authors explicitly note subject-specific overfitting and the need for larger, more diverse cohorts and multi-site validation before clinical deployment [83].

#### 5.2.2. How Label Delay and Label Noise Should Shape Validation

Wearable labels are often delayed, noisy, or indirect. Many outcomes are confirmed later by a reference test (e.g., patch review) or rely on self-report or ecological momentary assessment, and sleep staging depends on alignment to a reference scoring process. These realities should determine both the split strategy and the safeguards used. When labels are sparse or delayed, *temporal splits* are particularly important because they reduce the chance that closely neighbouring windows around a labelled event appear in both training and testing, which can inflate performance in autocorrelated streams. In addition, window extraction and any normalisation, imputation, or SQI threshold selection should be performed *after* splitting to avoid leakage of subject-specific or session-specific statistics into the test set. Where label timing is uncertain, studies should state the alignment rule (e.g., onset versus midpoint, tolerance window) and report sensitivity analyses over plausible alignment windows, rather than assuming perfect synchrony. Finally, when label noise is substantial, it is often safer to adopt an explicit deferral or referral policy (abstain when quality or confidence is low), and to report the resulting coverage and workload alongside performance on accepted cases [31,33,77].

#### 5.2.3. Metrics Beyond AUROC

AUROC is often insensitive to performance differences in highly imbalanced tasks and can hide an unacceptable false-alert burden. For rare events, precision–recall summaries (AUPRC and precision at fixed recall) are usually more informative. Sleep and actigraphy validation studies commonly report epoch-by-epoch confusion matrices and agreement analyses, because the clinical value depends on errors over time, not only aggregate discrimination [31]. For screening or alarm tasks, report event-based measures such as episode sensitivity, false alerts per day, and time-to-detection. For regression tasks (for example, cuffless blood pressure), report error distributions and agreement measures that reflect clinical interpretation rather than only mean absolute error.

Where streaming outputs are smoothed or aggregated (for example, averaging logits over a window), the evaluation should report both the instantaneous performance and the decision-level performance after aggregation, since aggregation can reduce momentary noise but can also delay detection [23]. In addition, metrics should be stratified by context (rest versus movement), by signal quality bands, and by device tier (device-only versus phone-gateway) when this affects the pipeline.

#### 5.2.4. Common Evaluation Errors and How to Avoid Them

Three errors recur in wearable modelling.

First, *leakage via preprocessing and window overlap*. When windows overlap heavily, adjacent segments can be near duplicates. Randomly splitting at the window level can therefore inflate results because the test set contains segments that are effectively seen during training. This is especially easy to miss in streaming pipelines where windowing and buffering are central design choices [23]. Subject-wise, time-based, or session-based splitting should be applied before window extraction where possible.

Second, *overfitting to subject, device, or collection routine*. Wearables capture stable personal traits and device-specific artefacts. Strong internal performance can therefore reflect memorisation of participants or sensor idiosyncrasies. Reporting dispersion across users (not only a mean) and testing on unseen devices or cohorts helps reveal this failure mode [24,83].

Third, *non-representative activity contexts*. Many models are trained on controlled protocols and then fail in free-living settings dominated by motion artefact and variable wear compliance. Papers should report the activity distribution, the unusable segment rate, and how the system behaves when quality is poor. Field validation is the most direct remedy, but where field studies are not feasible, stress tests should simulate realistic noise and missingness patterns and report performance by context [20,33].

### 5.3. Reliability Toolkit

Reliability in wearable edge systems is not a single metric. It is the combination of (i) well-calibrated outputs that support safe thresholds and deferral, (ii) monitoring that detects when conditions have changed, and (iii) update practices that preserve safety when models, firmware, or upstream pipelines evolve. The aim is to move from “a score” to an operational policy that is stable under free-living artefacts and lifecycle change. Table 5 summarises the reliability components, common methods, recommended metrics, and minimum reporting items for bedside-ready wearable ML systems.

#### 5.3.1. Calibration and Uncertainty for Action (Thresholds, Abstention, and Referral)

For bedside use, the question is often not “which class”, but “how safe is this decision right now”. Calibration makes this explicit. Your model can look strong on aggregate, yet be poorly calibrated for the positive class or unstable across runs, which can translate into unreliable rule-in behaviour [85]. In wearable AF classification, Monte Carlo dropout produced competitive discrimination while revealing that calibration quality can differ substantially by class, and that whole-test-set summaries may hide clinically important miscalibration [85]. This motivates reporting reliability diagrams and calibration errors (e.g., ECE or class-conditional calibration measures), not only AUROC or F1.

Once uncertainty is quantified, it can be turned into a clinical policy. Selective classification is a simple and practical pattern: defer high-uncertainty cases to manual review and automate only the remainder. In a prehospital ACS example, uncertainty was operationalised using posterior predictive entropy, with a cutoff chosen to keep a target coverage (e.g., 80%) while improving rule-in and rule-out performance on the retained subset [86]. The key translation step is to define what “defer” means (repeat measurement, request confirmatory modality, or human review), and to report the resulting coverage and workload, not only improved performance on the accepted cases.

More structured triage policies are also emerging. The SA-ROC framework, for example, frames clinical reliability targets (such as PPV constraints for rule-in and rule-out) as explicit safety requirements and converts uncertainty outputs into static decision zones (automated rule-out, automated rule-in, and manual review) [87]. A useful feature in this line of work is that it surfaces the operational cost of safety, for instance by quantifying the size of the gray zone and the implied deferred workload [87]. In wearables, this is directly relevant to deployment planning because deferral rates can be high when motion and contact artefacts dominate.

#### 5.3.2. Drift Monitoring Triggers and What to Watch

Wearable systems drift for reasons that are often mundane. Sensor contacts degrade, wear patterns change, and firmware or vendor algorithms update. In sleep monitoring, even “research grade” devices are not immune: firmware and hardware changes can alter upstream activity count generation, and manufacturer changes to algorithms can make previously validated results non-comparable across time points [88]. This matters because validation is frequently reported without firmware and software versioning, yet those details can nullify earlier evidence [88].

Accordingly, drift monitoring should be anchored to triggers that are observable without waiting for adjudicated outcome labels. A pragmatic approach is to monitor in three layers: system integrity, performance, and impact. In a health-system monitoring framework, system integrity covers end-to-end uptime, data retrieval failures, and output delivery failures; performance monitoring tracks statistical behaviour and subgroup stability over time; impact monitoring checks whether downstream workflows still deliver value and do not introduce harm [89]. Even if your wearable pipeline is not embedded in an EHR, the same structure applies: integrity corresponds to sensing and inference failures (missing channels, BLE dropouts, and late windows), performance corresponds to changes in calibration and alert rates, and impact corresponds to alert follow-through and clinician or user burden.

Practically, your drift dashboard should include at least the following: unusable-window rate (by SQI band), distribution shifts in core features, alert rate per day, calibration drift (ECE or Brier), and performance stratified by device model and wear context where available. It is also reasonable to require persistence, for example, consecutive out-of-band periods, before triggering costly interventions such as retraining or algorithm rollback [89].

#### 5.3.3. Safe Update Strategies (Models, Firmware, and Governance)

For wearables, updates are part of life. The goal is not to avoid updates, but to make them reversible, testable, and transparent. In embedded and IoT settings, robust OTA approaches include version control with separation of development and production tracks, integrity checks, and rollback-capable partitions to recover safely from partial failures [71]. These concepts map cleanly to wearable ML: your deployment should specify how you stage releases, how you detect failures, and how you revert.

From a regulatory and governance perspective, Predetermined Change Control Plans (PCCPs) formalise this mindset by requiring that planned modifications are bounded, risk-based, evidence-based, and transparent, with a total product lifecycle perspective [90]. These principles are compatible with good ML practice, including representative data, separation of training and test sets, clinically relevant testing conditions, and post-deployment monitoring [90]. Importantly, patching and update logistics have real operational costs in healthcare organisations. Organisational studies of connected medical device patching highlight that manual updates do not scale and that remote updating is not always adopted, partly due to workflow disruption and inventory complexity [72]. For wearable deployments, this reinforces why updates must be designed to minimise disruption, and why reporting versioning, rollout strategy, and rollback is not optional if the system is intended for long-term monitoring.

#### 5.3.4. A Concise Checklist for Bedside-Ready Wearable ML

To keep claims comparable across papers, we recommend reporting the following minimum items alongside standard performance metrics:

- Calibration: reliability diagram; Brier score and at least one calibration error metric (ECE or class-conditional equivalent), plus threshold selection rationale [85].
- Uncertainty policy: explicit deferral rule (abstain, request confirmatory signal, or manual review) and the resulting coverage and workload [86,87].
- Context sensitivity: performance and deferral rates stratified by signal quality and activity context, and by device model where relevant [88].
- Monitoring plan: integrity metrics (missingness, uptime, and pipeline failures), performance drift indicators, and an action protocol with review cadence [89].
- Update governance: model and firmware versioning, staged rollout, integrity checks, and rollback strategy [71,90].

**Table 5:** Reliability toolkit for bedside-ready wearable ML systems: components, methods, metrics, and minimum reporting.

Reliability Component	Methods	Metrics	Reporting Minimum
Calibration	Post-hoc calibration; context-specific recalibration for flagged cohorts	Brier; ECE; calibration curves	Reliability diagram; Brier and ECE (or class-conditional calibration) and threshold selection rationale [85]
Uncertainty and deferral	Selective classification; triage zones (rule-in, rule-out, review)	Coverage; false alerts per day; PPV/NPV at operating points	Define deferral action and report coverage and workload implications [86,87]
Drift monitoring	Integrity, performance, impact monitoring; persistence screens for alerts	Unusable window rate; alert-rate drift; calibration drift	Monitoring cadence, triggers, responsible owner, and concrete actions [89]
Firmware and algorithm stability	Version pinning or “freeze” for studies; benchmark old vs new	Versioned comparisons; comparability checks	Firmware, OS, and algorithm versions; statement of how updates are handled [88]
Safe update governance	OTA with staged rollout; integrity validation; rollback partitions	Update success rate; rollback rate; update latency (where relevant)	Versioning scheme, staged rollout plan, integrity checks, rollback strategy [71,90]

## 6. Open Challenges and Conclusions

Despite rapid progress in wearable sensing, streaming inference, and on-device optimisation, bedside adoption remains uneven. The main reason is that real-world deployment exposes failure modes that are weakly expressed in curated datasets: sparse and fragmented data, unstable signal quality, delayed or noisy labels, shifting device ecosystems, and workflow constraints. In this final section, we synthesise the most persistent blockers and translate them into a short research agenda that is tractable for computer scientists working at the intersection of biosignals, streaming systems, and clinical use.

### 6.1. Key Barriers to Bedside Adoption

#### 6.1.1. Generalisation Under Real-World Data Sparsity and Fragmentation

A recurring barrier is that training and evaluation data do not match how devices are used in daily life. Even large cohorts may contain short, relatively continuous recordings that differ from real-world patterns of intermittent wear, charging gaps, and variable engagement. In addition, many large-scale wearable programmes provide aggregated summaries rather than raw, continuous signals, limiting algorithm development and external validation. Data are also fragmented across proprietary ecosystems, and are often not paired with validated clinical outcomes, which restricts the ability to demonstrate clinical utility across populations and settings [91]. This combination of sparse data, limited ground-truth linkage, and ecosystem fragmentation makes it difficult to claim transportability beyond the original study context.

#### 6.1.2. Artefacts and Acquisition Variability as a Primary Failure Mode

Wearable biosignals are highly sensitive to acquisition conditions. Electrode or sensor contact, motion, muscle activity, ambient interference, and operator skill (for handheld imaging such as POCUS) introduce variability that can dominate the physiological signal of interest. These artefacts are not merely a preprocessing nuisance: they change the effective input distribution and can induce brittle behaviour when models are deployed outside controlled protocols [91]. This is most visible for PPG and wearable ECG, where contact and motion artefacts can inflate unusable windows and destabilise downstream decisions, but it affects all modalities through different mechanisms.

#### 6.1.3. Labels, Ground-Truth Delay, and the Cost of Annotation

Many wearable tasks have delayed or weak labels. Community screening problems may require confirmatory testing, while stress and behavioural endpoints often rely on self-reports that are incomplete or inconsistent. This creates a mismatch between dense signal streams and sparse labels, and it inflates both variance and the risk of overfitting to cohort-specific routines. Label-efficient approaches can reduce this burden. For example, semi-supervised stress monitoring has been studied explicitly to mitigate incomplete or inaccurate self-reports in daily life, using label propagation and representation learning to make use of large unlabelled segments [92]. However, label efficiency alone is not sufficient: studies must still report how labels were generated, when they occur relative to the stream, and what uncertainty remains.

#### 6.1.4. Personalisation Benefits and Personalisation Risk

Personalisation is attractive because wearable signals vary strongly by person, device, and wear context. Yet personalisation also introduces new risks: calibration drift, hidden subgroup failure, and a tendency to tune to a specific device family or behavioural routine. The barrier is not that personalisation is impossible, but that it must be bounded by evidence and by operating policies that reflect prevalence and downstream burden. In low-prevalence screening, even strong sensitivity and specificity can yield poor positive predictive value and overwhelm confirmatory testing pathways, which motivates tailored thresholds and sequential screening strategies that reduce false positives and referral burden [91]. Personalisation strategies should therefore be evaluated not only on discrimination, but also on workload impact and false-alert burden under realistic prevalence.

#### 6.1.5. Monitoring Burden, Usability, and Workflow Integration

A system that performs well offline can still fail at the bedside if it increases workload, produces excessive false alerts, or is difficult to operate. Evidence from continuous monitoring with deterioration alerting highlights recurring usability barriers: connectivity and battery limitations, alarm management and alarm fatigue, patient comfort issues, training gaps, and workflow integration challenges [93]. Importantly, front-line clinicians, often nurses, are the primary users and operational owners of these systems, yet clinician-centred evaluations are frequently underrepresented [93]. This makes monitoring and governance a core adoption challenge, not an optional add-on.

#### 6.1.6. How Barriers Differ by Modality and Application Bucket

Although the blockers are cross-cutting, their dominant form varies by modality and use case:

- Cardio screening and arrhythmia monitoring (ECG/PPG):most sensitive to motion and contact artefacts, and most exposed to low-prevalence false positives in community screening; confirmatory pathways and sequential screening are often necessary [91].
- BP and haemodynamics (PPG and related surrogates): strongly affected by calibration, posture, and physiological confounding; personalisation may help, but requires careful governance and re-calibration.
- Sleep and respiration (PPG, accelerometry, and temperature): label quality and temporal alignment are central because reference standards are costly; device ecosystem changes can undermine comparability over time.
- Movement and falls (IMU): signal quality is often more stable than optical sensing, but wear location and adherence drive distribution shift; the main hazard is false alarms versus missed events in free-living use.
- Stress and affect (EDA, HRV, and multimodal): ground truth is the limiting factor; label noise and missing self-reports motivate semi-supervised and weakly supervised designs [92].

#### 6.1.7. Most Actionable Research Gaps for Computer Scientists

We highlight five gaps that are both technically concrete and directly tied to bedside adoption:

1. Context-aware quality modelling as a first-class system component: models and pipelines that treat signal quality and wear context as explicit inputs, and that expose unusable-window rates and failure modes by context [91].
2. Label-efficient learning with explicit temporal semantics: methods that exploit unlabelled streams while respecting label delay, sparse adjudication, and time alignment, with reporting that makes leakage hard to introduce [92].
3. Transportability across devices and ecosystems: benchmarks and protocols that require device-family splits, version reporting, and calibration-transfer tests, rather than single-device evaluation [91].
4. Workload-aware decision policies: operating-point selection that incorporates prevalence, false-alert burden, and referral pathways, including sequential screening and deferral policies that reduce unnecessary follow-up [91].
5. Monitoring that is operationally realistic: drift detection and governance designs that account for limited staff time, training needs, and usability constraints, with measurable alert burden and clear actions when performance changes [93].

#### 6.1.8. Concluding Remarks

The field has the sensing and modelling foundations to support streaming wearable monitoring, but bedside adoption depends on making reliability visible: evidence of transportability, robust handling of artefacts, defensible labels, bounded personalisation, and monitoring plans that respect workflow realities. A practical research programme in this area should therefore treat deployment constraints, usability, and evaluation design as core scientific variables rather than implementation details.

### 6.2. Practical Recommendations

This review highlights a recurring gap: many wearable and edge ML studies report strong offline accuracy, but leave readers unable to judge reproducibility, deployment realism, or clinical credibility. We therefore translate the preceding sections into two concise checklists, followed by a minimum viable pathway from a prototype to a pilot or field study. The items below are deliberately practical and intended to be used alongside established reporting and implementation checklists [94,95,96,97].

#### 6.2.1. Checklist for Authors: Reproducibility, Deployment Realism, and Translational Credibility

- State the claim and its boundaries. Define the clinical task, the decision point, the intended users, and the target population. Add a short constraints on generality statement: where the method is expected to work, and where it likely will not [95].
- Document data provenance and preprocessing end to end. Report sensor types, wear location, sampling rates, missingness handling, and any quality gating or artefact rejection. Specify the exact preprocessing sequence and ensure it is applied after data splitting to avoid leakage [94,95].
- Make labelling assumptions explicit. Describe label source(s), timing, delay, and noise (including how weak labels or self-reports were validated). If labels are asynchronous with the stream, specify the alignment rule and how you prevented “future” information from entering the input window [94,95].
- Report model specification in a reproducible form. Provide architecture, hyperparameters, optimisation settings, and the model selection procedure (including what was tuned, on which split, and with what stopping rule) [94,95].
- Use evaluation designs that match the deployment risk. Prefer subject-wise splits for generalisation to new individuals; add temporal splits for drift sensitivity; add device or site splits when cross-device or cross-context use is claimed [94,95].
- Report metrics that reflect clinical use. For rare events, prioritise precision–recall summaries and decision-threshold results. For event detection, report event-based scoring and time-to-detection or delay. Include calibration (e.g., Brier-type summaries and calibration curves) if outputs are used for thresholds or triage [94].
- Declare the deployment envelope. Provide the edge tier (device-only, device+phone), expected window size and update rate, latency budget per update, peak memory, model size, and energy or duty-cycle assumptions used in measurements [97].
- Release a reproducibility bundle. Share code, configuration files, exact package versions, random seeds, and evaluation scripts. If raw data cannot be shared, provide a clear access pathway or a representative subset plus synthetic or de-identified surrogates where appropriate [94,95].

#### 6.2.2. Checklist for Practitioners: Selection, Benchmarking, Monitoring, and Safe Updates

- Purpose and setting first. Specify why the wearable system is being used, where it will be used, and what success looks like in that service (monitoring, screening, or intervention support) [96].
- Define roles and responsibilities. Identify who sets up devices, who reviews outputs, who responds to alerts, and who maintains devices and data pipelines. Plan training and support materials for each role [96].
- Select metrics and devices based on population and wear site. Choose signals and derived metrics that are relevant to the clinical goal, and check validity in the intended population and wear location [96].
- Benchmark realistically. Measure latency per update, peak RAM, model size, and energy under realistic sampling rates and background load. Record firmware, OS, and app versions because they can change behaviour over time [96,97].
- Adopt an operational monitoring plan. Track (i) system integrity (data arrival, pipeline health, missing channels), (ii) performance drift (calibration and error patterns), and (iii) operational impact (downstream actions and workload). Define trigger thresholds and escalation routes [97].
- Plan safe updates. Use versioned releases, staged roll-outs, and rollback capability. Treat model updates as clinical changes: document what changed, why, and what was re-validated [97].

#### 6.2.3. Minimum Viable Pathway: Prototype to Pilot or Field Study

1. Problem framing and workflow mapping. Define the decision supported, the acceptable response time, and the failure modes that matter most. Pre-register the evaluation plan when feasible [95].
2. Data and labels with auditability. Build a small but representative dataset that includes expected activities and artefacts. Define label timing, adjudication rules, and exclusion criteria before modelling [94,95].
3. Offline modelling with deployment-matched splits. Train baselines and candidate models using subject-wise and, where relevant, device and temporal splits. Report calibration and threshold performance, not only ranking metrics [94].
4. Edge integration and benchmarking. Implement the full streaming pipeline (windowing, buffering, and missingness handling) and benchmark latency, memory, and energy in conditions close to use. Freeze and log software and firmware versions [96,97].
5. Shadow mode pilot. Run the system without influencing care at first, then with supervised use. Collect end-user feedback, quantify false alerts and workload, and verify that the model remains calibrated in practice [96].
6. Field deployment with monitoring and update governance. Move to a field study only after monitoring triggers, escalation routes, and rollback procedures are in place. Maintain an internal change log that links each update to re-validation evidence [97].

### 6.3. Future Directions

Several research threads are converging towards more reliable, resource-aware wearable machine learning. A practical goal is to move from narrow, one-task models to reusable representations that can be adapted across devices, settings, and endpoints. Recent surveys describe three broad routes: training biosignal-specific models at scale, adapting general time-series pretraining to biomedical signals, and using text-centric models to support signal analysis when paired reports are available [41]. Across all routes, the same engineering constraint remains: wearable deployment needs models that tolerate heterogeneous sampling rates, channel configurations, and noise, while still supporting clinically meaningful tasks. To provide clearer strategic value, we organise future directions into a staged roadmap spanning short-term achievable steps, mid-term system capabilities, and longer-term aspirational foundations.

#### 6.3.1. Short Term (1–2 Years): Make Claims Comparable and Deployment-Realistic

The most actionable near-term work is standardisation. Progress is currently slowed by uneven evaluation: models are assessed on different datasets, splits, and downstream definitions, which makes comparison unreliable [98]. The community would benefit from shared tasks and benchmark protocols that include device- and subject-shift splits, stress tests for missing channels and sensor noise, and reporting templates that bind performance claims to edge costs. Benchmark suites such as BiomedBench argue for end-to-end evaluation that reflects wearable duty cycles and phase-specific costs (idle, acquisition, and processing), rather than reporting only model size or desktop throughput [62]. A practical deliverable is a minimal benchmark card for wearable ML papers: input sampling assumptions, per-window latency, peak RAM, energy per inference (or per hour at a stated duty cycle), calibration checks, and at least one evaluation under device or subject shift. In parallel, quality-aware pipelines should be treated as first-class components, with explicit reporting of unusable-window rates and the effect of SQI gating on both coverage and false-alert burden.

#### 6.3.2. Mid Term (2–4 Years): Safe Personalisation and Continual Learning Under Constraints

Wearable systems face inter-subject variability and domain shift across device models, wear locations, and activity context. Personalisation can help, but it introduces safety and governance risks if updates are uncontrolled. Federated learning is attractive because it can train across sites without moving raw data, but heterogeneity and compute overhead are persistent barriers. FedEE illustrates a pragmatic direction by combining personalisation with uncertainty estimation and efficiency, using lightweight early-exit blocks to approximate ensembling within a single backbone [99]. Continual learning is the other half of the problem: models should adapt to new tasks or changing distributions without catastrophic forgetting. Hierarchical continual learning in federated settings has been proposed as one way to retain domain knowledge while adapting over time, combining ongoing learning with stabilisation techniques such as knowledge distillation and elastic weight consolidation [100]. For bedside translation, these methods should be coupled to explicit update policies (when to adapt, when to abstain, when to refer) and continuous calibration monitoring, rather than treating personalisation as a one-off fine-tune.

#### 6.3.3. Long Term (4+ Years): Edge Foundation Models with Safety and Budget Guarantees

The longer-term direction is edge-first biosignal foundation models that offer transferable representations while respecting deployment budgets and safety constraints. A key design tension is sequence length versus compute. Patch-based tokenisation reduces sequence length and cost but can blur boundary details; point-based inputs preserve high-frequency detail but are expensive. Attention-based models are widely used for representation learning, yet their scaling with sequence length can be limiting, motivating more efficient backbones such as state-space models for long continuous signals [98]. Large pre-trained models are already being built with heterogeneity in mind. CSFM learns unified representations across ECG, PPG, and associated text at large scale and reports transfer across diverse cardiac tasks and sensing scenarios [101]. AnyPPG uses synchronised PPG and ECG to learn physiologically grounded PPG representations from large volumes of unlabeled recordings, then evaluates generalisation across multiple downstream tasks [102]. For on-device use, the central research problem is not only accuracy but also the cost of inference and updates. Distillation and structured compression remain strong candidates because they preserve a stable student interface and can be repeated as teachers improve. PPG-Distill provides one example, combining prediction-, feature-, and patch-level objectives to transfer both global and local knowledge from larger PPG teachers into smaller students [103]. A key aspiration is to formalise “budgeted adaptation”: methods that adapt within a fixed latency and peak RAM envelope, with quantised deployment paths, rollback rules, and measurable calibration stability.

#### 6.3.4. Prioritised Research Gaps for Computer Scientists

- Benchmarks that bind claims to costs (short term): shared tasks plus standard edge metrics (latency, RAM, energy, and duty cycle) and reproducible measurement practice [62].
- Quality-aware streaming pipelines (short term): explicit SQI gating, missingness handling, and reporting of usable-window rates and false-alert burden under realistic activity contexts.
- Safety-aware personalisation (mid term): federated and continual learning designs with explicit uncertainty, calibration monitoring, and rollback rules for updates in the field [99,100].
- Edge-first foundation models (long term): pretraining objectives and architectures that handle heterogeneous channels and sampling while providing efficient on-device inference and adaptation [101,102,103].

## 7. Conclusions

Wearable biosignals, combined with edge machine learning, are increasingly able to support continuous health monitoring beyond the clinic. Across modalities such as ECG, PPG, IMU, and EDA, the practical value of edge deployment is clear: lower latency, reduced dependence on connectivity, and improved privacy by limiting transmission of raw signals. However, this review also shows that bedside translation depends less on novel architectures alone and more on whether systems are engineered and evaluated for the conditions they will face in daily life.

We synthesised an end-to-end view from sensing and streaming structure to model families, deployment optimisation, and tiered architectures. In the application landscape, rhythm monitoring and AF screening are comparatively mature due to clearer reference standards and growing ambulatory validation evidence, while cuffless blood pressure estimation, fine-grained sleep staging, and stress inference remain more sensitive to calibration drift, label uncertainty, and context confounding. Across all use cases, the dominant barriers are consistent: artefacts and acquisition variability, sparse and delayed ground truth, domain-shift across people and devices, personalisation risk, and the operational burden of monitoring and updates.

To support more reliable translation, we proposed practical validation and reliability guidance that prioritises subject-wise and device-aware evaluation, metrics beyond AUROC, calibration and uncertainty-aware operating policies, and post-deployment monitoring with safe update governance. Looking ahead, edge-first biosignal foundation models, budgeted adaptation, privacy-preserving personalisation, and standardised benchmarks with edge cost metrics offer a clear research agenda for computer scientists. Progress will be fastest when claims about clinical utility are paired with reproducible pipelines, transparent reporting, and field evidence that links model outputs to meaningful actions and manageable workload.

## Figures and Tables

**Table 1:** Core wearable modalities used in edge health monitoring, with typical sampling ranges, dominant artefacts, and common preprocessing or signal quality indices (SQIs). Sampling ranges are device dependent and shown as typical values reported across the wearables literature.

Modality	Sampling	Common Artefacts	Typical Preprocessing/SQI
ECG (dry, wet, capacitive)	125–500 Hz [1,12]	Motion artefact; contact loss; baseline wander; impedance changes; mains interference [17,18,19]	Band-pass + baseline removal; QRS/beat detection; SQI from beat agreement, SNR, morphology consistency, and segment usability flags [13,17]
PPG (wrist, finger)	25–200 Hz [12,20]	Motion artefact; ambient light; contact pressure variation; low perfusion; sensor displacement [13,20]	Detrending + band-pass; motion reduction (e.g., adaptive filtering); SQI using pulse-shape checks, perfusion measures, SNR, and artefact screens before HR/SpO_2_ [13,20]
IMU (accelerometer, gyroscope)	25–200 Hz [1,15]	Placement variability; orientation changes; bias/drift; saturation/clipping; packet loss [15]	Calibration and gravity removal; orientation normalisation; dropout/saturation checks; quality checks via range, stationarity, and plausibility of derived gait/activity features [15]
EDA (skin conductance)	4–32 Hz [14,21]	Motion spikes; electrode hydration and skin-impedance shifts; sweat effects; thermal/environment confounding [14,21]	Low-pass smoothing + despiking; tonic/phasic separation; SQI from contact stability, spike rate, and plausibility of tonic/phasic dynamics [14,21]
Skin temperature	0.1–1 Hz (often slower) [1,14]	Loose contact; ambient temperature shifts; slow sensor drift; contact transients [14]	Smoothing and outlier rejection; context-aware interpretation; quality checks for contact stability and abrupt non-physiologic jumps [14]

Abbreviations: ECG—electrocardiography; PPG—photoplethysmography; IMU—inertial measurement unit; EDA—electrodermal activity; SQI—signal quality index; SNR—signal-to-noise ratio.

**Table 2:** Model families for streaming biosignals at the edge, summarising typical strengths, limitations, edge suitability, and common modalities or tasks.

Model Family	Strengths	Limitations	Edge Suitability	Typical Modalities/Tasks
Feature-based + compact classifiers	Physiological interpretability; low compute; stable latency; simple to audit [44,46]	Depends on robust detectors; brittle under artefacts; may miss subtle morphology and longer context [13,46]	High	ECG rhythm features; PPG HR/HRV; IMU activity features [47]
1D CNN (incl. depthwise separable)	Efficient local pattern learning; good with short windows; compresses well [8,37]	Limited long-range context unless stacked/dilated; can be sensitive to sensor and subject shift [46]	High	ECG beat/rhythm; PPG waveform learning; IMU window classifiers [46,47]
TCN/dilated CNN	Larger receptive field at modest cost; stable training; streaming-friendly with fixed windows [8]	Still window-bound; variable-length events need careful windowing; dilation choices matter [8]	High–Medium	ECG/PPG sequences; IMU gait and activity sequences [8]
RNN family (GRU/LSTM)	Captures temporal dependence across windows; supports variable-length sequences [47]	Less parallelism; higher energy and latency; compression can be harder [8]	Medium	IMU activity; forecasting; some ECG rhythm pipelines [47]
Efficient attention/Transformer encoders	Flexible context integration; masking supports missing channels; useful for multimodal fusion [41]	Compute and memory can be high; streaming needs careful design to avoid repeated attention [41]	Medium–Low	Multimodal wearables; longer-context ECG; cross-device representations [41]
Hybrid CNN + attention (or CNN + Transformer)	CNN captures morphology cheaply; attention adds context; often a good trade-off when tuned [38]	More design choices; must report edge cost and validation clearly [38]	Medium	ECG arrhythmia where context helps; compact hybrid edge models [38]
Self-supervised pretrained encoder + small head	Reduces label needs; transferable representations; efficient heads can suit edge [41,42]	Pretraining mismatch can hurt; encoders may be large; reliability gains need verification [41]	Medium	ECG embeddings for low-label tasks; single-lead wearable ECG adaptation [42,43]

Abbreviations: CNN—convolutional neural network; TCN—temporal convolutional network; RNN—recurrent neural network; GRU—gated recurrent unit; LSTM—long short-term memory.

**Table 3:** Recommended deployment levers for streaming biosignal models, with typical benefits, risks, and best-use scenarios.

Technique	Expected Gains (Size/Latency/Energy)	Common Risks/Failure Modes	Best-Use Cases (Biosignals)
Post-training int8/full-int8 quantisation	Large size reduction; often meaningful latency and energy reduction when integer kernels are supported [40]	Calibration mismatch can degrade accuracy; unsupported “hybrid” paths on some microcontroller runtimes; layer sensitivity (notably early feature extractors) [40]	CNN/TCN-style ECG or PPG classifiers; rhythm detection where predictable latency is required
Quantisation-aware training (QAT)	Better accuracy retention than post-training quantisation in difficult cases; improved robustness to quantisation noise	Higher training burden; requires careful alignment of training-time fake quant with deployment kernels	When post-training quantisation causes unacceptable performance drops (e.g., subtle morphology tasks in ECG/PPG)
Structured pruning (channels/filters)	Can reduce latency and energy if the resulting dense model is smaller; improves cache behaviour	Over-pruning harms generalisation; pruning can interact with artefact sensitivity in short windows [48]	Conv models for ECG/PPG where compute is dominated by convolution blocks; on-device continuous monitoring
Unstructured pruning (weight sparsity)	Reduces storage; may reduce energy if sparse kernels are available	Often limited latency gains on standard runtimes; sparse kernel support varies widely	Storage-constrained deployments where model size is the limiting factor
Knowledge distillation (teacher → student)	Enables smaller students with competitive performance; can improve small models versus training from scratch [49]	Student may inherit teacher biases; needs careful evaluation across devices and populations	PPG heart rate estimation; small on-device regressors/classifiers when labels are limited or noisy
Graph-level acceleration (operator fusion, vendor SDKs, runtime delegates)	Improves latency by reducing overhead and memory traffic; stabilises real-time throughput	Results depend on toolchain versions; hard to reproduce unless configs are released	Any streaming pipeline with frequent updates (short windows, high overlap), especially when CPU overhead dominates

**Table 4:** Use-case landscape for edge wearable monitoring: typical signals, model families, edge tier, and key deployment pitfalls.

Use Case	Signal(s)	Typical Model Family	Edge Tier	Key Pitfalls/Constraints
AF screening and burden	PPG (+ECG confirm)	Quality-gated features + classifier; compact 1D CNN/TCN	Watch + phone	Motion artefacts and unusable windows; ectopy-driven false positives; clinician review burden; demographic/device shift [77,78]
Arrhythmia monitoring (general)	ECG/PPG	Feature pipelines (HRV/morphology) or compact RNN/TCN	Device-only or device + phone	Battery versus sampling rate; alert fatigue; threshold calibration; update-induced drift [78]
Cuffless BP estimation	PPG (±ECG)	Regression/GBM; compact CNN/TCN; personalised calibration layers	Watch + phone	Calibration drift; posture and physiological confounding; validation under dynamic states; reporting of reference standard [79,80]
Sleep staging (epoch)	ACC + PPG + Temp	Feature-based + classifier; sequence models (TCN/RNN)	Watch + phone	PSG alignment and epoch definition; proprietary algorithms; label noise; limited access to raw epochs [81]
Fall detection	IMU	Thresholding + compact NN (CNN/Transformer)	Device + phone gateway	Wear location and user compliance; BLE/gateway range assumptions; false alarms versus missed events; battery constraints [82]
Frailty/functional gait assessment	IMU	Time-series transforms + classifier; compact models	Device-only	Subject-specific effects; need for larger external validation; long-term wearability and comfort [83]
Stress/affect inference	HRV + EDA (+Temp/ACC)	Multimodal fusion; personalised models	Phone-edge (often)	Confounding by activity and environment; uncertain ground truth; weak labels; limited linkage to clinical endpoints [84]

## Data Availability

No new data were created or analyzed in this study.
